# Feasibility, Drug Safety, and Effectiveness of Etiological Treatment Programs for Chagas Disease in Honduras, Guatemala, and Bolivia: 10-Year Experience of Médecins Sans Frontières

**DOI:** 10.1371/journal.pntd.0000488

**Published:** 2009-07-07

**Authors:** Oliver Yun, M. Angeles Lima, Tom Ellman, Wilma Chambi, Sandra Castillo, Laurence Flevaud, Paul Roddy, Fernando Parreño, Pedro Albajar Viñas, Pedro Pablo Palma

**Affiliations:** 1 Médecins Sans Frontières/Doctors Without Borders, New York, New York, United States of America; 2 Médecins Sans Frontières, Operational Center Barcelona-Athens (OCBA), Barcelona, Spain; 3 Médecins Sans Frontières/Médicos Sin Fronteras, La Paz, Bolivia; 4 Laboratory of Parasitological Diseases, Oswaldo Cruz Institute, FIOCRUZ, Rio de Janeiro, Brazil; Universidad de Buenos Aires, Argentina

## Abstract

**Background:**

Chagas disease (American trypanosomiasis) is a zoonotic or anthropozoonotic disease caused by the parasite *Trypanosoma cruzi*. Predominantly affecting populations in poor areas of Latin America, medical care for this neglected disease is often lacking. Médecins Sans Frontières/Doctors Without Borders (MSF) has provided diagnostic and treatment services for Chagas disease since 1999. This report describes 10 years of field experience in four MSF programs in Honduras, Guatemala, and Bolivia, focusing on feasibility protocols, safety of drug therapy, and treatment effectiveness.

**Methodology:**

From 1999 to 2008, MSF provided free diagnosis, etiological treatment, and follow-up care for patients <18 years of age seropositive for *T. cruzi* in Yoro, Honduras (1999–2002); Olopa, Guatemala (2003–2006); Entre Ríos, Bolivia (2002–2006); and Sucre, Bolivia (2005–2008). Essential program components guaranteeing feasibility of implementation were information, education, and communication (IEC) at the community and family level; vector control; health staff training; screening and diagnosis; treatment and compliance, including family-based strategies for early detection of adverse events; and logistics. Chagas disease diagnosis was confirmed by testing blood samples using two different diagnostic tests. *T. cruzi*-positive patients were treated with benznidazole as first-line treatment, with appropriate counseling, consent, and active participation from parents or guardians for daily administration of the drug, early detection of adverse events, and treatment withdrawal, when necessary. Weekly follow-up was conducted, with adverse events recorded to assess drug safety. Evaluations of serological conversion were carried out to measure treatment effectiveness. Vector control, entomological surveillance, and health education activities were carried out in all projects with close interaction with national and regional programs.

**Results:**

Total numbers of children and adolescents tested for *T. cruzi* in Yoro, Olopa, Entre Ríos, and Sucre were 24,471, 8,927, 7,613, and 19,400, respectively. Of these, 232 (0.9%), 124 (1.4%), 1,475 (19.4%), and 1,145 (5.9%) patients, respectively, were diagnosed as seropositive. Patients were treated with benznidazole, and early findings of seroconversion varied widely between the Central and South American programs: 87.1% and 58.1% at 18 months post-treatment in Yoro and Olopa, respectively; 5.4% by up to 60 months in Entre Ríos; and 0% at an average of 18 months in Sucre. Benznidazole-related adverse events were observed in 50.2% and 50.8% of all patients treated in Yoro and Olopa, respectively, and 25.6% and 37.9% of patients in Entre Ríos and Sucre, respectively. Most adverse events were mild and manageable. No deaths occurred in the treatment population.

**Conclusions:**

These results demonstrate the feasibility of implementing Chagas disease diagnosis and treatment programs in resource-limited settings, including remote rural areas, while addressing the limitations associated with drug-related adverse events. The variability in apparent treatment effectiveness may reflect differences in patient and parasite populations, and illustrates the limitations of current treatments and measures of efficacy. New treatments with improved safety profiles, pediatric formulations of existing and new drugs, and a faster, reliable test of cure are all urgently needed.

## Author Summary

Chagas disease was discovered 100 years ago by the Brazilian physician Carlos Chagas. Predominantly affecting poor populations throughout Latin America, recognition and treatment of this parasitic disease are often neglected. Since 1999, the international medical humanitarian aid organization Médecins Sans Frontières (Doctors Without Borders) has offered diagnostic and therapeutic care for Chagas disease, and here we describe four of our programs in Honduras, Guatemala, and Bolivia, 1999–2008. The earliest programs focused on treating young children and in subsequent programs expanded up to 18 years of age. We identified six program components essential for project viability: information, education, and communication; vector control; health staff training; screening and diagnosis; treatment and compliance; and logistics. The number of children and adolescents screened for Chagas disease ranged from over 7,500 to nearly 25,000 in each program. Early analysis of cure rates ranged widely: from 87% and 58%, respectively, in Honduras and Guatemala, to 0%–5% in Bolivia. No deaths occurred in any of the programs, though drug-related side effects were observed in a quarter to half of all patients. Through our findings and experience, we discuss the feasibility, safety, and effectiveness of treatment programs for Chagas disease in resource-limited settings.

## Introduction

Discovered 100 years ago in 1909, Chagas disease (American trypanosomiasis) is an endemic disease of the Americas, caused by infection with the protozoan parasite *Trypanosoma cruzi*. According to varying estimates, there are about 10–15 million existing cases, 50,000 new annual infections, and 14,000 deaths per year [1]–[5]. Chagas disease primarily affects populations in low-income, resource-poor areas, where health care is often lacking or difficult to access.

The first initiatives for controlling Chagas disease focused primarily on prevention through vector control and screening of blood donors, but with limited resources directed towards diagnosing, treating, and following up those already infected either during or after vector control activities. In 1999, the international medical humanitarian organization Médecins Sans Frontières/Doctors Without Borders (MSF) started its first program for the diagnosis and treatment of Chagas disease for affected populations.

Through its Spanish, French, and Belgian sections, MSF implemented six Chagas disease diagnosis and treatment programs in Honduras, Nicaragua, Bolivia, and Guatemala, from 1999 to 2008, focusing on pediatric populations [6]. In addition to its field programs, MSF helped develop information, education, and communication (IEC) modules in Argentina, Colombia, and Ecuador, and together with the Pan American Health Organization (PAHO) produced a virtual medical training course for the diagnosis and treatment of Chagas disease [7],[8].

Since 1999, MSF has treated over 3,100 patients for Chagas disease. Here we describe four programs run by MSF Operational Centre Barcelona Athens (OCBA) in 1999–2008 in endemic areas of Honduras, Guatemala, and Bolivia. We discuss the feasibility of implementing such projects in resource-limited settings in remote rural areas, through the analyses and validation of shared programmatic components, drug safety, and treatment effectiveness.

## Methods

### Settings and Populations

In collaboration with national health ministries, MSF implemented Chagas disease diagnosis and treatment programs in three rural districts and one periurban setting from 1999 to 2008. The rural programs were in Yoro, Honduras (latitude 15.3, longitude −87.1) from 1999 to 2002; Olopa, Guatemala (14.6, −89.3) from 2003 to 2006; and Entre Ríos, O'Connor Province, Tarija, Bolivia (−21.5, −64.7) from 2002 to 2006. The periurban program was in Sucre, Bolivia (−19.0, −65.2) from 2005 to 2008. All four programs were in areas with relatively poor populations who had limited access to medical care, with the rural areas in remote, difficult-to-access locations.

All four programs focused on pediatric and adolescent patients, but with an increase in age group treated over time: Yoro, <12 years old; Olopa and Entre Ríos, <15 years; and Sucre, <18 years. The increase in treatment age groups over time and projects reflected MSF's strategy to first diagnose and treat young children and then expand these services to older children and adolescents.

These areas were selected for medical intervention based on available *T. cruzi* seroprevalence information from national Chagas disease programs and preliminary seroprevalence surveys by MSF, presence of active vector control programs (indoor and peridomestic residual spraying and entomological surveillance), and limited health care access. Requirements for opening these programs included health care structures (eg, clinics, laboratories, offices, etc), equipment, supplies, and human resources at the primary health care level for carrying out laboratory serodiagnosis and storage of serum samples, together with immediate operational capacity for adequate diagnoses, treatment, and follow-up. MSF helped provide equipment; contracted additional temporary healthcare, logistical, and administrative staff, when needed; and bought all necessary supplies. All the programs had close interaction with national, departmental, and municipal programs regarding vector control activities, entomological evaluation and surveillance, and health education.

### Essential Program Components

All the Chagas disease diagnosis and treatment projects shared six principal, essential features directly assuring program feasibility in remote, rural settings (Table 1):

**Table 1:** Essential program components of Médecins Sans Frontières Chagas disease diagnosis and treatment projects in Central and South America, 1999–2008.

Program Component	Elements/Description	Goals
Information, education, and communication (IEC)	Learn local reality/situation through reports and direct contacts and establish dialogue with key leaders and actors; informational meetings targeting four main groups: community authorities, health staff, key community figures, and patient families; focused sessions on diagnosis and treatment (follow-up, adverse events, among others); informed consent forms; community feedback meetings	Design correct IEC approach based on local socioeconomic contexts; provide insight into cultural constraints and health-seeking behaviors; raise public awareness of the disease and of availability of diagnosis/treatment services
Vector control	Precondition for treatment in national Chagas programs, with varying levels of MSF involvement, but always with participation/follow-up of information and surveillance systems	National program collaboration; disease prevention; prevention of re-infection
Health staff training	Chagas-oriented training for health personnel	Establish family commitment to treatment and follow-up
Screening and diagnosis	Active screening at community level; filter paper for testing in Yoro, Olopa, and Entre Ríos; Chagas Stat-Pak RDT (whole blood) introduced in Entre Ríos and Sucre	Identify patients for treatment; optimize diagnostic protocols
Treatment and compliance	Inclusion criteria: cut-off ages (expanded over time/projects), acute or recent chronic disease phase (indeterminate form); populations within catchment areas, informed consent; exclusion criteria: pregnant or lactating women, renal or hepatic impairment, severe and/or generalized disease, drug hypersensitivity; follow-up by doctors on treatment initiation and adverse events, and by nurses on compliance; defaulters to follow-up actively traced; importance of serological follow-up emphasized; follow-up reinforcement messages; family-based strategy for compliance and adverse event monitoring	Ensure treatment completion; monitor adverse events; proper follow-up care; family engagement
Logistics	Physical, geographical access to remote communities; monitoring and evaluation of vector control; use of community structures for meetings, IEC sessions, training, screening, and follow-up	Access to patients; delivery of care to remote areas

1. Information, education, and communication (IEC): The four main target audiences were community authorities, health staff, key community figures (eg, teachers, religious leaders, etc), and patient families. The first step was to learn about the local reality and situation through published and reported information and through direct contacts, and second step was to establish a dialogue with different leaders and actors to design the correct IEC approach taking into account socioeconomic and cultural contexts. In each program, meetings to spread IEC messages within the four target populations were held before screening activities. A more focused IEC session on diagnosis and treatment (especially on follow-up and adverse events) was given to families prior to patient detection, confirmation, and treatment. Informed consent forms signed by the patient's family were compulsory before inclusion in each program. After treatment, community meetings were held to obtain feedback on activities.

2. Vector control: As a precondition for treatment in national programs for Chagas disease, MSF involvement in vector control activities was adapted to each country's situation and capacity. In Yoro, Honduras, MSF was directly involved in vector control activities; in the programs in Guatemala and Bolivia, vector control was directed by the national programs. Before starting diagnosis and treatment of patients, a prerequisite was that infestation rates of the given community had to be <3% following national protocols. If a child was found to be seropositive, the family's house was checked to be vector-free, with targeted spraying if needed. Entomological surveillance in the community (Puesto de Informacion de Vinchucas [PIV], or Vector Information Post) was regularly performed.

3. Training of health staff: Chagas disease-oriented training of health staff members was carried out for teaching specific diagnosis and treatment skills, and how to communicate and work with patient families to help ensure adherence and follow-up and for early adverse event detection and rapid intervention.

4. Active screening and diagnosis: Active disease screening at the community level was implemented in all four projects. In each program, the whole population found in the municipality based on target patient age group was screened. Different diagnostic guidelines were established in the four projects depending on agreed-upon protocols, field availability of tests, feasibility of implementation, and expert technical advice. Screening and diagnosis were implemented at the primary health care level.

5. Treatment and compliance, including family-based strategies: Inclusion criteria for etiological treatment in all programs were enrollment of children of different cut-off age groups, with age groups expanded in newer programs as programmatic experience and evidence were gained; patients in acute or recent chronic phase (indeterminate form) regardless of transmission route; populations within the catchment area of the project; and signed, informed consent by parents or guardians. Exclusion criteria included pregnant and lactating women; patients with renal or hepatic impairment or failure; any severe or generalized disease; and drug hypersensitivity.

Some weekly follow-up sessions were handled by doctors, focusing on treatment initiation and addressing adverse reactions, while the remainder of follow-ups were handled by nurses focused on treatment compliance. When adverse events were unmanageable, a referral system including third-level hospitals was used, with referred patients followed up on a daily basis. Defaulters to follow-up visits were actively traced by health staff or community health workers. Serological follow-up was emphasized among patients at the time of initial result, with serum samples taken before treatment initiation. Reinforcement messages for treatment adherence were given with full course of treatment. All care was provided free of charge.

Treatment and compliance included a family-based strategy in which parents and guardians of patients were co-responsible for daily drug administration, early detection of adverse events, and requesting medical help for patient treatment withdrawal, if needed.

6. Logistics: Logistic activities focused on access to remote communities and close monitoring and evaluation of vector control measures. The supply chain for drugs and laboratory reagents was maintained, as was storage of frozen samples for serological testing. Community structures, such as schools, were used for relevant activities, including community meetings, IEC sessions, training, screening, and treatment follow-up. MSF worked in close collaboration with national Chagas disease programs in terms of logistics in all four projects.

### Screening and Diagnosis

According to World Health Organization (WHO) recommendations, diagnosis of Chagas disease was confirmed using two different tests. In case of doubtful or discordant results, a third test was used. Following national and regional recommendations, each project used different tests, as follows.

Diagnostic testing for *T. cruzi* was performed by ELISA (conventional and recombinant), indirect hemagglutination (HAI), and, for exceptional confirmation needs, indirect immunofluorescence (IFI). The source of reagents for ELISA was Wiener or Biochile.

In Yoro and Olopa, screening was conducted using conventional ELISA using filter paper. Confirmation of diagnosis was done with recombinant ELISA. Similarly, in Entre Ríos, conventional ELISA and HAI tests were conducted, with recombinant ELISA as the tiebreaker. When necessary, IFI was used instead of HAI. Later in the Entre Ríos program, Chagas Stat-Pak (Chembio Diagnostic Systems, Inc, Medford, NY) rapid diagnostic test (RDT) was introduced for screening, using whole blood samples, and all positive results were systematically confirmed by conventional ELISA and HAI, and recombinant ELISA used as a tiebreaker. In Sucre, screening was conducting using Chagas Stat-Pak on whole blood. As in Entre Ríos, positive results were confirmed using conventional ELISA and HAI, with tiebreakers assessed via recombinant ELISA.

For conventional and recombinant ELISA, cut-off values were calculated according to manufacturer recommendations by taking the sum of the absorbance of all negative controls and adding this to a constant factor (0.200 for conventional, 0.300 for recombinant). Positive results were those samples with an optic deviation (DO) above cut-off+10%. Negative results were those with DO below cut-off−10%. Doubtful results were those with DO between (cut-off−10%) and (cut-off+10%). For HAI, positive results were those samples with reactivity for dilution ≥1/16 titration. Positive reactions for dilutions at 1/2, 1/4, or 1/8 were considered cross-reactive and false-positive; protocol called for these samples to be treated with 2-mercaptoethanol 1% and HAI repeated. For Chagas Stat-Pak RDT, positive results were those samples giving two pink/purple lines, one in test area and one in control area, at reading at 15 minutes (maximum 30 minutes). Tests with no line visible in the control area were considered invalid, and these samples were retested using a new device.

Quality control (QC) measures were systematically performed in the programs. For RDT QC, for every 10^th^ negative RDT result, venous blood was taken and sent to the laboratory for ELISA/HAI testing. For ELISA/HAI QC, internal QC was performed using the positive and negative controls present in the test kit (and a performance checklist was also used for QC on the procedure itself). Overall, 10% of positive samples and 10% of negative samples were sent to the reference laboratory for external QC.

### Treatment and Follow-up

*T. cruzi*-positive patients were treated with benznidazole 5–7.5 mg/kg/day, 2 or 3 times per day over 60 days (maximum 300 mg/day; if necessary, the total dose was calculated and divided for more than 60 days). In the four programs, counseling for the parents/guardians of infected children as provided, informing them of how to give treatment, potential treatment benefits, risk factors, and adverse events, including how to proceed if adverse events occur. Treatment and follow-up (at days 0, 7, 14, etc) were provided by health staff, while daily drug tablets were administered at home by the parenets/guardians. Treatment adherence sheets were filled out by parents/guardians or patients. In results analysis, a patient was considered as having completed treatment when >30 days of treatment were accomplished.

Passive, and when necessary, active, weekly patient follow-up was performed in all projects by physicians or nurses. When necessary, more intensive and/or more frequent follow-up was performed. Clinical presentation and adverse events were recorded.

### Adverse Events

The severity of adverse events was recorded at each follow-up visit. Adverse events were classified as mild, moderate, or severe. Mild adverse events were defined as those requiring no treatment interruption. Moderate adverse events were defined as those requiring temporary treatment interruption, with the patient returning to treatment within 14 days. Severe adverse events were defined as those requiring treatment stoppage. All adverse events were evaluated by a physician, and symptomatic treatment was given according to their type and severity. The types of adverse events observed were as follows: dermatological, gastrointestinal, and neurological.

### Seroconversion

To assess seroconversion from positive to negative for *T. cruzi* infection, the first post-treatment serologic evaluation was generally conducted at 18 or 36 months post-treatment. Post- and pre-treatment blood samples were processed simultaneously using conventional ELISA. Negative results from conventional ELISA were confirmed with recombinant ELISA. All ELISA tests used serum or plasma samples. All pre-treatment samples (serum/plasma) were aliquoted and frozen (without glycerin) at −20°C, less than 24 hours after collection. All ELISA test results were obtained using an ELISA reader (optical density visible in the reader screen).

Based on WHO protocol, cure was defined as two non-reactive ELISA tests (one conventional, one recombinant) performed on the same sample on the same date. For patients with positive or indeterminate results in the first evaluation, a second serology evaluation was generally performed at 36 months post-treatment.

### Statistical Analyses

Normalized differences in antibody titers were calculated in consecutive assessment comparisons to pre-treatment baseline values by using the following equation: (final antibody titers−initial antibody titers)/initial antibody titers)×100. Likewise, differences in *T. cruzi* antibody titers between pre-treatment baseline and post-treatment control values were compared using Wilcoxon ranked sum test, and negative seroconversion rates between 18 and 36 months after treatment were compared using McNemar test. Mann-Whitney U-test and Kruskal-Wallis test were used to compare differences in *T. cruzi* antibody titers, while Chi-square or Fisher's exact test were used to analyze negative seroconversion and tendency to seroconversion rates according to age and gener. 95% confidence intervals for rate differences were calculated. Statistical significance was set at 5%. All tests of significance were two-tailed.

### Ethics

Informed written consent was obtained before treatment from parents or guardians of patients who tested positive. If parents/guardians were illiterate, oral explanation was given, and consent was obtained by fingerprint. All data were collected routinely and managed confidentially. All the projects were discussed, reviewed, and approved by the national Ministry of Health (MOH), with MOH permission granted before starting each program.

## Results

### Program Summaries

#### Yoro, Honduras, 1999–2002

MSF opened its first Chagas disease treatment program in 1999, in the rural district of Yoro, Honduras. The program diagnosed and treated those <12 years of age. Vector control activities were carried out directly by MSF in this program. In December 2002, the program was handed over to the local health authorities.

From 1999 to 2002, 24,771 patients were tested for *T. cruzi* infection (Table 2). Of these, 232 children were positive, giving a seroprevalence of 0.9%. All diagnosed and treated children were asymptomatic with indeterminate form, except for one child who presented an acute infection with Romaña's sign (unilateral periorbital edema).

**Table 2:** Chagas disease patient diagnosis and treatment results of four Médecins Sans Frontières programs in Central and South America, 1999–2008.

	Yoro (Honduras)	Olopa (Guatemala)	Entre Ríos (Bolivia)	Sucre (Bolivia)
Program duration	1999–2002	2003–2006	2002–2006	2005–2008
Age group (years)	<12	<15	<15	<18
# patients tested	24,771	8,927	7,613	19,400
# patients positive at initial screening	256	124	1,475	1,179
# patients confirmed positive/infected	232	124	1,475	1,145
Seroprevalence (%)	0.9	1.4	19.4	5.9
# patients treated	231	124	1,409	1,040
Seroconversion rate (%)	87.1	58.1	5.4	0

A total of 231 patients (9 months to 12 years of age) was treated with benznidazole. All patients received a full 60-day course of treatment except for 1 acute case treated with a 30-day protocol, 3 cases in which treatment was stopped (at 43, 49, and 50 days) due to adverse events, and 3 cases treated for almost a full course (53, 54, and 56 days of treatment). Since a 30-day treatment is considered correct for acute cases, 98.7% (228/231) of patients completed treatment.

#### Olopa, Guatemala, 2003–2006

In 2003, MSF started a Chagas disease management program for patients <15 years old in the rural area of Olopa, Guatemala. From 2003 to 2006 (end of MSF involvement), 8,927 children were screened, with 124 found to be positive for *T. cruzi* infection, resulting in an estimated seroprevalence of 1.4% in the department of Olopa (Table 1). All diagnosed and treated children were asymptomatic in the indeterminate form.

Of the 124 infected patients, 123 were treated with benznidazole. Of those started on treatment, 95% completed a full course of therapy (58–60 days). Thirteen (10.5%) patients interrupted treatment, but only 4 (3.2%) stopped treatment.

#### Entre Ríos, Bolivia, 2002–2006

MSF opened its first Chagas disease program in Bolivia in 2002. In a rural area of Tarija department, a highly endemic region for Chagas transmission, the program aimed to treat all children <15 years old.

A total of 7,613 children was screened, of whom 1,475 were confirmed with *T. cruzi* infection, giving a seroprevalence rate of 19.4% (Table 1). Seroprevalence by age group was 5.0% in <5 years old; 14.8% in the age group 5–9 years old; 31.0% 10–14; and 51.7% 15–16. Of these, 1,409 patients began treatment, and 1,363 completed at least 30 days of treatment and 1,276 completed at least 55 days (standard course of treatment). A total of 28 (2%) children stopped treatment due to adverse events.

#### Sucre, Bolivia, 2005–2008

In 2005, MSF started Chagas disease diagnosis and treatment in two districts of Sucre, a periurban environment in Bolivia. Building upon previous programmatic experience and striving to increase care to more patients with Chagas disease, the age group for treatment was expanded up to 18 years old. In addition to expanding the treatment age group, this program was unique in its introduction of RDTs as a diagnostic screening tool, based on Bolivian national protocol (diagnosis with Chagas Stat-Pak RDT and confirmation with ELISA). In 2007, the opportunity was taken to perform a field evaluation of the use of whole blood with Chagas Stat-Pak RDT [9].

A total of 19,400 children was tested in two districts of Sucre, of whom 1,145 were positive for *T. cruzi*, resulting in a seroprevalence rate of 5.9% (Table 1). Seroprevalence by age group was 1.9% <5 years old; 4.1% 5–9; 8.6% 10–14; and 14.2% 15–18. Of these, 1,040 patients started benznidazole treatment. A total of 912 (87.7%) patients completed a full course of treatment, 18 of whom completed after switching to nifurtimox (second-line treatment) due to adverse events. A total of 61 (5.8%) patients (0% in <5 years old; 8.6% 15–18) stopped treatment due to adverse events.

### Adverse Events

Drug safety was assessed by recording treatment-related adverse events in terms of severity and type. In all four programs, most adverse events were mild. No deaths due to treatment occurred in any of the programs.

In the Central American programs in Yoro, Honduras and Olopa, Guatemala, 50.2% and 50.8% of patients, respectively, had adverse events related to treatment (Table 3). In Yoro, most of the adverse events were mild, with no moderate cases and 3 severe cases due to neurological adverse events (neuromuscular disturbances of the lower limbs after 6 weeks of treatment). The most frequent adverse events were gastrointestinal disorders (26.8%, mainly epigastralgia and/or abdominal pain, and less frequently nausea and/or vomiting and anorexia), followed by dermatological conditions (13.0%, mainly pruritus and less frequently maculopapular exanthema) and neurological problems (10.4%, mainly neuromuscular disturbances). In Olopa, 80.9% (51/63) of the adverse events were mild, 14.3% (9/63) moderate, and 4.8% (3/63) severe (2 neuromuscular and 1 cutaneous). Adverse events were 26% dermatological in nature, 25% gastrointestinal, 23% neuromuscular, and 26% other types. In both Yoro and Olopa, no differences were seen in the proportion of adverse events depending on age or sex (Chi-square test).

**Table 3:** Benznidazole-related adverse events in four Médecins Sans Frontières Chagas disease treatment programs in Central and South America, 1999–2008.

Program	Adverse Events				
	Number of patients	% of all patients treated	Mild, %	Moderate, %	Severe, %
Yoro, Honduras	116	50.2	97.4	0	2.6
Olopa, Guatemala	63	50.8	80.9	14.3	4.8
Entre Ríos, Bolivia	361	25.6	79.5	18.8	1.7
Sucre, Bolivia	394	37.9	61.4	28.2	10.4

Lower rates of treatment-related adverse events were observed in the Bolivian programs. In Entre Ríos, adverse events were observed in 25.6% of treated children, with increasing risk in older age groups (12% in <5 years old; 25% 10–14). In Sucre, 37.9% of patients had adverse events, also with increasing risk in older groups (13.4% in <5 years old; 50% 15–18). In Entre Ríos, 56% of adverse events were dermatological, 25% digestive, and 18% neuromuscular, of which 11% were mixed. In Sucre, 68.5% of adverse events were dermatological. In both programs, the majority of side effects were mild, with risk increasing with age.

Six and 41 severe adverse events were reported in Entre Ríos and Sucre, respectively. In these two programs, 1 case of Lyell syndrome (toxic epidermic necrolysis) and 1 case of Stevens Johnson syndrome were reported. Lyell syndrome occurred in a 13-year-old girl at day 34 of benznidazole treatment. In the weekly follow-up, the patient showed a generalized itchy rash with good general clinical status and was treated with oral antihistamine drugs. Two days later, a MSF physician was contacted and visited the child, who presented with high fever and general cutaneous rash with infected pustules. The patient was given intravenous fluids and ceftriaxone until admission to Tarija hospital. She was managed and discharged after 7 days with good clinical improvement.

### Seroconversion Rates

Treatment effectiveness was measured by rates of seroconversion in the patients. A marked difference was seen in the rates of seroconversion between patients treated in the two earlier Central American programs (Yoro, Olopa) compared with the two later programs in South America/Bolivia (Entre Ríos, Sucre).

In Yoro, Honduras, seroconversion rate for *T. cruzi* was 87.1% (202/232) at 18 months post-treatment, showing a high seroconversion rate achieved in a relatively short period of time (Table 1). At 36 months, seroconversion rate was 92.7% (215/232). In Olopa, Guatemala, from available patient data (25.5% of the treatment cohort), seroconversion at 18 months post-treatment was 58.1% (18/31).

Seroconversion rates observed in Entre Ríos and Sucre in Bolivia were much lower. Preliminary results of overall seroconversion post-treatment was 5.4% (59/1,101) in Entre Ríos by up to 60 months post-treatment, with over 950 of the patients sampled having had follow-up later than 18 months post-treatment. Seroconversion rates were found to be lower in older age groups compared with younger ones in Entre Ríos: 24.2% (16/66) <5 years old; 4.6% (14/303) 5–9 years old; 1.9% (12/638) 10–14 years old, at 18–60 months follow-up. To date, of 276 patients followed up between 9 and 27 months post-treatment, no patient has been found to have seroconverted in Sucre.

## Discussion

The 10-year operational experience of MSF in these four programs in Honduras, Guatemala, and Bolivia demonstrates that diagnosis and treatment of Chagas disease are feasible, relatively safe, and potentially effective in low-income, resource-constrained settings. Through the lessons learned from earlier studies [10],[11] and these MSF projects and their common, essential logistical components, we propose that this programmatic approach is feasible at the primary health care level and replicable in other Chagas-disease endemic countries and regions, even in periurban and remote rural areas. With proper coordination between different stakeholders focused on integrated health care services for Chagas disease, including national and regional programs, the diagnosis and treatment of the disease in early chronic phases (mainly indeterminate form) can be safely implemented and should be deemed necessary for affected populations [12],[13].

Etiological treatment of Chagas disease can and should be integrated at the primary health care level because most patients are near primary health care services, and the majority of patients would be able to receive medical care at this level, taking into account the proportion of Chagas patients with the indeterminate form of the disease. In MSF's programs, this implementation was achieved in remote rural settings through the application of six central features and criteria: IEC, vector control, health staff training, logistics, screening/diagnosis, and treatment/compliance, with family-based support.

IEC was a chief component of program strategies and is vital to ensure treatment compliance and early detection of adverse events, especially when providing care for populations with differing cultures, practices, and modes of communication, among others. IEC was crucial for raising awareness in the general population about the disease (regarding transmission routes, clinical manifestations, and treatment and prevention possibilities) and inform patients and patient families that diagnosis and treatment services were available.

Vector control carried out by national programs was also an important program component and should be simultaneously implemented with patient access to diagnosis and treatment [14],[15]. MSF involvement varied as projects progressed, depending on the need and capacity of national authorities and other partners. After treatment, vector control was continued through the national programs, but regular spraying every 6 months was not always carried out. Community entomological surveillance occurred regularly, but spraying for vector control was irregular at times. Eliminating the vector from the environment and households of patients and those at risk is critical.

Health staff training and family IEC for family-based treatment monitoring were exceptional ways of both ensuring quality of diagnosis and treatment compliance, as well as engaging the family in the health care process. Diagnosis and treatment of Chagas disease in all the projects relied on well-trained health personnel to apply their medical skills to care for patients and to establish family commitment to treatment adherence and follow-up care. With minimum logistical capacity, especially support for outreach teams, our program experience may be replicable in other endemic areas.

The fundamental program component of screening and diagnosis used differing diagnostic protocols adapted to the contexts of each country/region. For diagnosis in our programs, the two tests selected were the two with minimum acceptable sensitivity and specificity (ideally 99–100%) and which could be feasibly implemented at the primary health care level [6],[16]. Filter paper blood samples were used in the earlier programs mainly for sensitivity and adapted ease of use (ie, no need for centrifugation, relatively easy to supply/refill, portability) in remote rural settings. We introduced the use of Chagas Stat-Pak RDT in the Bolivian programs and carried out a field evaluation using whole blood samples. Recent studies using this RDT have shown relatively low sensitivity (93–94%) compared with conventional tests [9],[17],[18], and this limited sensitivity must be considered in the use of this test. A whole-blood RDT with high sensitivity would be ideal for screening and diagnosis in resource-limited settings [19].

For treatment and compliance, a large number and proportion of patients started and finished treatment according to protocol in our programs, with over 90% of patients completing >55 days of treatment. Access to treatment, follow-up, and referral of complicated cases were successful elements of the protocol. Relatively low dropout before treatment and low default rates (mostly migrations of patients and adverse reactions) were observed. However, in one program, Sucre, about 9% of diagnosed patients did not start treatment. The main reasons for this were migration, reluctance to start treatment (after counseling and informed consent), pregnant or lactating mothers, and treatment being offered by MOH national programs. Still, overall we found that Chagas treatment and follow-up can be achieved with adequately trained, sensitized, and motivated health staff and family members in both rural remote and periurban settings. The family-based approach for daily drug administration and compliance was key for Chagas disease because of the length of therapy and occurrence of adverse events.

Drug treatment was safely administered in these four programs, with no deaths occurring due to adverse events. Despite this, nearly half of all patients had some type of adverse event, a few of which were severe, including 1 case of Lyell syndrome, and 1 case of Stevens Johnson syndrome. Although no previous studies of Chagas disease have reported either of these syndromes, these two cases must be viewed in the context of over 3,000 patients treated in the four programs, with no deaths in even the most severe cases. The majority of adverse event cases were treated with a reduced dosage of benznidazole (to the minimum dose of 5 mg/kg/day) or temporary suspension of treatment. The time of appearance, intensity, and clinical patterns of adverse events were not different than those observed in other experiences [20], except that we did not see any hematological reactions (ie, no clinical manifestations such as anemia, severe infection, or hemorrhage were observed to make us suspect detrimental effects on bone marrow). However, hematological reactions were only followed clinically, without routine laboratory testing, due to issues of practicality under field conditions. This therefore poses a limitation in that hematological adverse events cannot be completely excluded, especially since severe hematological reactions (such as bone marrow suppression) can be asymptomatic. Proximal neuromuscular adverse events presented later (after 35 days of treatment) compared with other adverse event types, demonstrating cumulative drug toxicity.

Overall, the large number of children and adolescents treated and observed in the four programs (>3,100) provides valuable insight into drug safety for current Chagas disease drug treatment. Previous studies have reported experiences from lower numbers of patients [10],[21]. Of note, we observed sizeable variations in reported adverse events in the study locations, namely between the two programs in Central America (Honduras/Guatemala) and the two in South America (Bolivia). In recording adverse events and their severity in our four programs, observer bias no doubt played a role. The identification and classification of an adverse event is often dependent on the observing medical staff, and misclassifications were possible in the programs. We attempted to address this by defining mild, moderate, and severe adverse events based on whether treatment was temporarily interrupted or fully stopped. Other biases in adverse event profiles may exist, such as differences in early detection of side effects and more or less intensive medication and management for adverse events.

While a well-designed program should be able to minimize risks and ensure safe treatment, the lack of a non-toxic alternative drug remains a major obstacle to wider access to treatment for both adults and children. No pediatric formulation currently exists for benznidazole (nor nifurtimox, the only other drug used for treating Chagas disease), increasing risks of under- or overdosing in children. For the youngest patients, cutting tablets and mixing with water or other liquids for oral administration is difficult and has important pharmacological implications in terms of absorption and bioavailability.

The seroconversion rates detected in treated patients were relatively high in the Central American projects, Yoro, Honduras and Olopa, Guatemala, showing that therapy can clear *T. cruzi* infection. However, seroconversion was far lower in the South American Bolivian projects in Entre Ríos and Sucre. The findings in Bolivia are similar to those reported from earlier studies in Argentina and Brazil [22]–[25]. Also, seroconversion was detected earlier in the Central American programs compared with the Bolivian programs. Previous research has shown that in South America seroconversion is sometimes not detected until 5–7 years later [26]. Thus, the higher and earlier seroconversion we detected in Central America supports previously reported findings [27] and may have important public health implications.

The differences in seroconversion rates may be explained by a number of reasons. One primary explanation may be based on the presence of different parasite lineages in different geographic regions, with *T. cruzi* type I predominating in Central America and *T. cruzi* type II in South America, with varying degrees of overlap [28]. Because of the potential differences in *T. cruzi* subtypes present in Honduras and Guatemala compared with Bolivia, drug treatment effectiveness may have differed. Another factor to consider is the time between vector control activity and drug treatment, since cases (mostly asymptomatic) closer to the acute phase of the disease can possibly account for more rapid seroconversion. Also, statistical limitations of our data analysis may exist due to the varying age groups and varying times of post-treatment follow-up in the four projects, as has been examined in other studies [29]. Finally, differences in immune response among populations may play a role.

Whatever the reason, the lack of a better marker for indicating parasitological cure is a major impediment to advances in treatment and development of more effective drugs [4]. The observed differences between seroconversion rates in Central and South America highlight the need for further studies to confirm our findings and help improve etiological treatment protocols with dosages and duration adapted to the Chagas disease cycle in different geographic regions.

Since the start of our first Chagas disease program in 1999, which focused on young children, MSF has pushed to deliver diagnosis and treatment of this disease to wider and wider age groups. Over the past decade, treatment for Chagas disease has expanded from children <12 years old, to <15, then <18, and finally adults. This strategy has helped bring broader coverage of treatment delivery for Chagas disease.

Bolivia is the most highly endemic country in the world for *T. cruzi* infection, with up to 1.8 million people believed to be infected [1], [30]–[32]. MSF currently has two active programs in Cochabamba, where Chagas disease treatment is integrated into primary health care and offered to adults as well as children and adolescents. Because of high prevalence in Bolivia, Chagas disease diagnosis and treatment remain an operational priority there for MSF.

MSF's programs, both past and present, highlight where and what the needs are for people affected by Chagas disease. In addition to increasing public awareness and patient access to existing diagnostics and drugs, the development of new, less toxic, more effective drugs; adapted pediatric formulations of treatments; and a reliable test of parasitological cure are all urgently required. Because Chagas disease and those afflicted with it are often neglected, medical care for this patient population should be implemented whenever and wherever possible, as MSF has demonstrated as feasible in these four programs, and research and development for the disease should be scaled up dramatically.

## References

[1] WHO (2002). Control of Chagas disease: second report of the WHO expert committee.. http://whqlibdoc.who.int/trs/WHO_TRS_905.pdf.

[2] Remme JHF, Feenstra P, Lever PR, Medici A, Morel C (2006). Tropical diseases targeted for elimination: Chagas disease, lymphatic filariasis, onchocerciasis, and leprosy.. “Disease Control Priorities in Developing Countries”, 2^nd^ ed..

[3] World Health Organization (2004). World health report 2004: changing history. Deaths by cause, sex and mortality stratum in WHO regions, estimates for 2002. Annex Table 2.

[4] Tarleton R, Reithinger R, Urbina JA, Kitron U, Gurtler RE (2007). The challenges of Chagas disease – grim outlook or glimmer of hope?. PLoS Neglect Trop Dis.

[5] Pan American Health Organization Estimación cuantitativa de la enfermedad de Chagas en les Américas [Spanish]..

[6] Villa L, Morote S, Bernal O, Bulla D, Albajar-Vinas P (2007). Access to diagnosis and treatment of Chagas disease/infection in endemic and non-endemic countries in the XXI century.. Mem Inst Oswaldo Cruz.

[7] Pan American Health Organization Virtual medical training course in the diagnosis, management and treatment of Chagas disease.. http://www.paho.org/english/ad/dpc/cd/dch-curso-virtual-msf.htm.

[8] Pan American Health Organization (2005). PAHO/MSF Regional Technical Consultation on the Organization and Structure of Medical Care to Infected/Ill Patients by Trypanosoma cruzi/Chagas Disease. Montevideo, Uruguay, October 13–14th, 2005.. Revista da Sociedade Brasileira de Medicina Tropical.

[9] Roddy P, Goiri J, Flevaud L, Palma PP, Morote S (2008). Field evaluation of a rapid immunochromatographic assay for detection of *Trypanosoma cruzi* infection by use of whole blood.. J Clin Microbiol.

[10] de Andrade AL, Zicker F, de Oliveira RM, Almeida Silva S, Luquetti A (1996). Randomised trial of efficacy of benznidazole in treatment of early Trypanosoma cruzi infection.. Lancet.

[11] Sosa Estani S, Segura EL, Ruiz AM, Velazquez E, Porcel BM, Yampotis C (1998). Efficacy of chemotherapy with benznidazole in children in the indeterminate phase of Chagas' disease.. Am J Trop Med Hyg.

[12] Segura E (2002). El control de la enfermedad de Chagas en la República Argentina.. http://www.paho.org/Spanish/AD/DPC/CD/dch-historia-incosur.htm.

[13] Zaidenberg M, Spillman C, Carrizo-Paez R (2004). Control de Chagas en la Argentina. Su evolución.. Revista Argentina de Cardiología.

[14] Gürtler RE, Kitron U, Cecere MC, Segura EL, Cohen JE (2007). Sustainable vector control and management of Chagas disease in the Gran Chaco, Argentina.. Proc Natl Acad Sci U S A.

[15] Gürtler RE (2007). Combining residual insecticide spraying campaigns with targeted detection and specific chemotherapy for *Trypanosoma cruzi* infection in children.. PLoS Negl Trop Dis.

[16] Coura JR (2007). Organización y Estructura de la Atención Médica en la Infección/Enfermedad de Chagas. Lecciones aprendidas en 15 proyectos.. La enfermedad de Chagas. A la puerta de los 100 años del conocimiento de una endemia americana ancestral. (Silveira AC org.).

[17] Sosa-Estani S, Gamboa-León MR, Del Cid-Lemus J, Althabe F, Alger J (2008). Use of a rapid test on umbilical cord blood to screen for *Trypanosoma cruzi* infection in pregnant women in Argentina, Bolivia, Honduras, and Mexico.. Am J Trop Med Hyg.

[18] Chippaux JP, Santalla JA, Postigo JR, Romero M, Salas Clavijo NA (2009). Sensitivity and specificity of Chagas Stat-Pak(R) test in Bolivia..

[19] Technical Report (2008). New diagnostic tests are urgently needed to treat patients with Chagas disease meeting. 30–31 August 2007. Médecins Sans Frontières. Campaign for Access to Essential Medicines. Rio de Janeiro, Brazil.. Revista da Sociedade Brasileira de Medicina Tropical.

[20] Coura JR, Castro SL (2002). A critical review on Chagas disease chemotherapy.. Mem Inst Oswaldo Cruz.

[21] Cançado JR (2002). Long term evaluation of etiological treatment of chagas disease with benznidazole.. Rev Inst Med Trop Sao Paulo.

[22] Fernandes CD, Tiecher FM, Balbinot MM, Liarte DB, Scholl D, Steindel M, Romanha A (2009). Efficacy of benznidazol treatment for asymptomatic chagasic patients from state of Rio Grande do Sul evaluated during a three years follow-up.. Mem Inst Oswaldo Cruz.

[23] Streiger ML, del Barco ML, Fabbro DL, Arias ED, Amicone NA (2004). Longitudinal study and specific chemotherapy in children with chronic Chagas' disease, residing in a low endemicity area of Argentina[Portuguese].. Rev Soc Bras Med Trop.

[24] Schijman AG, Altcheh J, Burgos JM, Biancardi M, Bisio M (2003). Aetiological treatment of congenital Chagas' disease diagnosed and monitored by the polymerase chain reaction.. J Antimicrob Chemother.

[25] Sosa-Estani S, Segura EL (2006). Etiological treatment in patients infected by Trypanosoma cruzi: experiences in Argentina.. Curr Opin Infect Dis.

[26] Luquetti AO, Rassi A (2002). Conferencia: Perspectiva del uso de la serología (Ag naturales y otros) en la evaluación de la eficacia del tratamiento etiológico.. http://www.fac.org.ar/fec/chagas2/llave/c003/luque.htm.

[27] Guhl F, Nicholls RS, Montoya R, Rosas F, Velasco F (2004). Rapid negativization of serology after treatment with benznidazole for Chagas disease in a group of Colombian schoolchildren. Proceedings of the IX European Multicolloquium of Parasitology, Valencia (Spain), July 18–23, 2004. Articles of Keynote Speakers. Volume I ISBN 88-7587-115-9.. Medimond International.

[28] Recommendations from a Satellite Meeting (1999). International Symposium to commemorate the 90th anniversary of the discovery of Chagas disease, April 11–16 1999, Rio de Janeiro, Brazil.. Mem Inst Oswaldo Cruz.

[29] Sosa-Estani S, Herrera de Bravo B, Herrera de Bizzoto L, Canil S, Cura EN, Segura EL (2002). Evolución serológica a largo plazo en niños infectados por Trypanosoma cruzi que cursan fase clínica indeterminada, tratados con Benznidazol.. http://www.fac.org.ar/fec/chagas2/llave/md8/md804/sosaes.htm.

[30] Guillén G (2002). El control de la enfermedad de Chagas en Bolivia.. http://www.paho.org/portuguese/ad/dpc/cd/dch-historia-incosur.pdf.

[31] Schmuñis GA, Brener Z, Andrade ZA, Barral-Netto M (2000). A Tripanossomíase Americana e seu Impacto na Saúde Pública das Americas.. Trypanosoma cruzi e Doença de Chagas. 2^nd^ ed.

[32] Valencia A (1990). Investigación epidemiológica nacional de la Enfermedad de Chagas..

